# Modeling Caribou Movements: Seasonal Ranges and Migration Routes of the Central Arctic Herd

**DOI:** 10.1371/journal.pone.0150333

**Published:** 2016-04-05

**Authors:** Kerry L. Nicholson, Stephen M. Arthur, Jon S. Horne, Edward O. Garton, Patricia A. Del Vecchio

**Affiliations:** 1 Department of Fish and Wildlife Sciences, University of Idaho, Moscow, Idaho, United States of America; 2 Alaska Department of Fish and Game, Division of Wildlife Conservation, Fairbanks, Alaska, United States of America; University of Alaska Anchorage, UNITED STATES

## Abstract

Migration is an important component of the life history of many animals, but persistence of large-scale terrestrial migrations is being challenged by environmental changes that fragment habitats and create obstacles to animal movements. In northern Alaska, the Central Arctic herd (CAH) of barren-ground caribou (*Rangifer tarandus granti*) is known to migrate over large distances, but the herd’s seasonal distributions and migratory movements are not well documented. From 2003–2007, we used GPS radio-collars to determine seasonal ranges and migration routes of 54 female caribou from the CAH. We calculated Brownian bridges to model fall and spring migrations for each year and used the mean of these over all 4 years to identify areas that were used repeatedly. Annual estimates of sizes of seasonal ranges determined by 90% fixed kernel utilization distributions were similar between summer and winter (*X̅* = 27,929 SE = 1,064 and *X̅* = 26,585 SE = 4912 km^2^, respectively). Overlap between consecutive summer and winter ranges varied from 3.3–18.3%. Percent overlap between summer ranges used during consecutive years (*X̅* = 62.4% SE = 3.7%) was higher than for winter ranges (*X̅* = 42.8% SE = 5.9%). Caribou used multiple migration routes each year, but some areas were used by caribou during all years, suggesting that these areas should be managed to allow for continued utilization by caribou. Restoring migration routes after they have been disturbed or fragmented is challenging. However, prior knowledge of movements and threats may facilitate maintenance of migratory paths and seasonal ranges necessary for long-term persistence of migratory species.

## Introduction

Migration is a distinctive characteristic of many animals, and large-scale migrations are found worldwide among ungulate species such as wildebeest (*Connochaetes taurinus*), plains zebra (*Equus quagga*) and elephants (*Laxodonta africana*) in Africa [1], red deer (*Cervus elephus*) and moose (*Alces alces*) in Europe, saiga antelope (*Saiga tatarica*) in central Asia [2], and pronghorn (*Antilocapra americana*), bison (*Bison bison*), and elk (*Cervus elaphus*) in North America [3] [4]. Animals migrate to obtain seasonally-available resources [5], avoid adverse weather, give birth or raise young in areas where predation risk is reduced [6], or avoid overcrowded conditions [7, 8]. Although migration has been defined in various ways [9, 10], we consider migration to be the periodic (e.g., annual) movement away from and subsequent return to a similar location [3, 11]. Migratory behavior results in 2 or more distinct areas of frequent use (i.e., seasonal ranges) that are at least partially separated by a matrix of areas of little or no use. Routes used by animals to travel between seasonal ranges (hereafter, migration routes) have received considerable attention in recent years due to concerns about maintaining connectivity between seasonal ranges of imperiled species or populations [2–4]. Restoring disturbed migration routes can be challenging. However, better knowledge of animal movements and potential threats to mobility may enable protection measures to be adopted before migration routes become obstructed or fragmented [4, 12]. Identifying areas used for migration by terrestrial mammals is a critical step in long-term management of these species.

In North America, many populations of caribou (*Rangifer tarandus*) are known or suspected to make seasonal migrations [13–17]. Caribou migration routes are often located in areas where shallow or hard snow or frozen rivers and lakes facilitate travel [18–21], and adverse snow conditions may delay migration by hindering caribou movements [22]. To accommodate spatial variability in environmental conditions, natural selection has likely favored caribou that follow migration routes that proved successful during previous years [22]. In such cases, young caribou may learn by following older, experienced animals [23]. Such reliance on traditional migration routes might delay or reduce the ability of caribou to adapt to environmental changes.

North American caribou are commonly assigned to herds, based on the traditional use of seasonal ranges, particularly areas used during the calving season [24–26]. In northern Alaska, 4 herds of barren-ground caribou (*R*. *t*. *granti*) have been identified. Recent (2008–2011) estimates of abundance of these herds were 55,000 for the Teshekpuk Lake herd, 67,000 for the Central Arctic (CAH), 169,000 for the Porcupine herd, and 348,000 for the Western Arctic herd (Alaska Department of Fish and Game, Fairbanks, AK, unpublished data).

Although seasonal migrations of the large arctic caribou herds are among the most spectacular movements exhibited by terrestrial mammals of North America, these migrations are poorly documented in the scientific literature. This may be due in part to the difficulty of characterizing movement patterns of large groups of animals, while acknowledging the temporal variability in these movements. For the CAH, previous research was mostly concerned with understanding the effects of oil development on local movements and habitat use. For example, several studies investigated the potential for disruption of movements and habitat use on the summer range as a result of construction of the Trans-Alaska Pipeline (TAP) and the associated oil fields along the Arctic coast of northeastern Alaska (e.g., [27–32]), and others have studied the effects of oil development on the location and use of calving ranges (e.g., [29, 33–35]). Several studies have presented detailed models of caribou calving ranges in northern Alaska (e.g., [36–38]) and Person et al. [39] described seasonal distributions of the Teshekpuk Lake herd. However, quantitative analyses of migration routes and range use by the CAH during summer and winter are lacking.

Despite these limitations, the general pattern of seasonal movements of caribou in northern Alaska has been described (e.g., [40, 41]). Migratory movements of the CAH are oriented principally north-south, extending from winter range in the mountains and foothills of the Brooks Range to summer and calving ranges on the tundra-dominated Arctic coastal plain [28, 30, 42, 43]. Spring migration occurs during April and May and is led by pregnant females, who may travel 7–24 km/day [44–46]. Males and non-pregnant females follow the pregnant females, and they join to form large groups after calving ends during mid-June [47]. Summer movements are likely driven by foraging requirements and by caribou responses to harassment by biting insects [48]. During the mid-summer peak of insect activity, caribou often gather in large, dense aggregations in windy areas, along the coast, or on persistent patches of snow or ice [27, 48]. During August, insect activity diminishes and caribou begin a slow and irregular movement southward to higher elevations [30, 41]. Fall migration (defined as movements directed toward wintering areas) usually begins during September and continues through November [24, 49, 50].

Identifying the geographic areas used by the CAH during different seasons is needed to determine effective conservation measures and guide planning for future development of roads and infrastructure related to extraction of oil, gas, minerals, and other resources in northern Alaska. In addition, differences in areas used between seasons or among years may suggest environmental forces that affect caribou movements. Conversely, consistency in use of a particular area, either across seasons or during the same season of different years, may indicate important habitats. Thus, assessing fidelity in range use may indicate important habitats or suggest directions for future studies. Our objectives were to develop models to estimate and quantify summer (post calving season) and winter ranges of the CAH; identify geographic areas used during spring and fall migrations; and assess annual variation in seasonal ranges and migratory movements. We also estimated several metrics describing timing, distance, and rate of movement during migration, which may be useful as baseline data or for comparison with other populations. We restricted our study to movements of adult female caribou, because caribou herd designations are generally based on movements and selection of calving areas by females.

## Materials and Methods

### Study area

**Fig 1:**
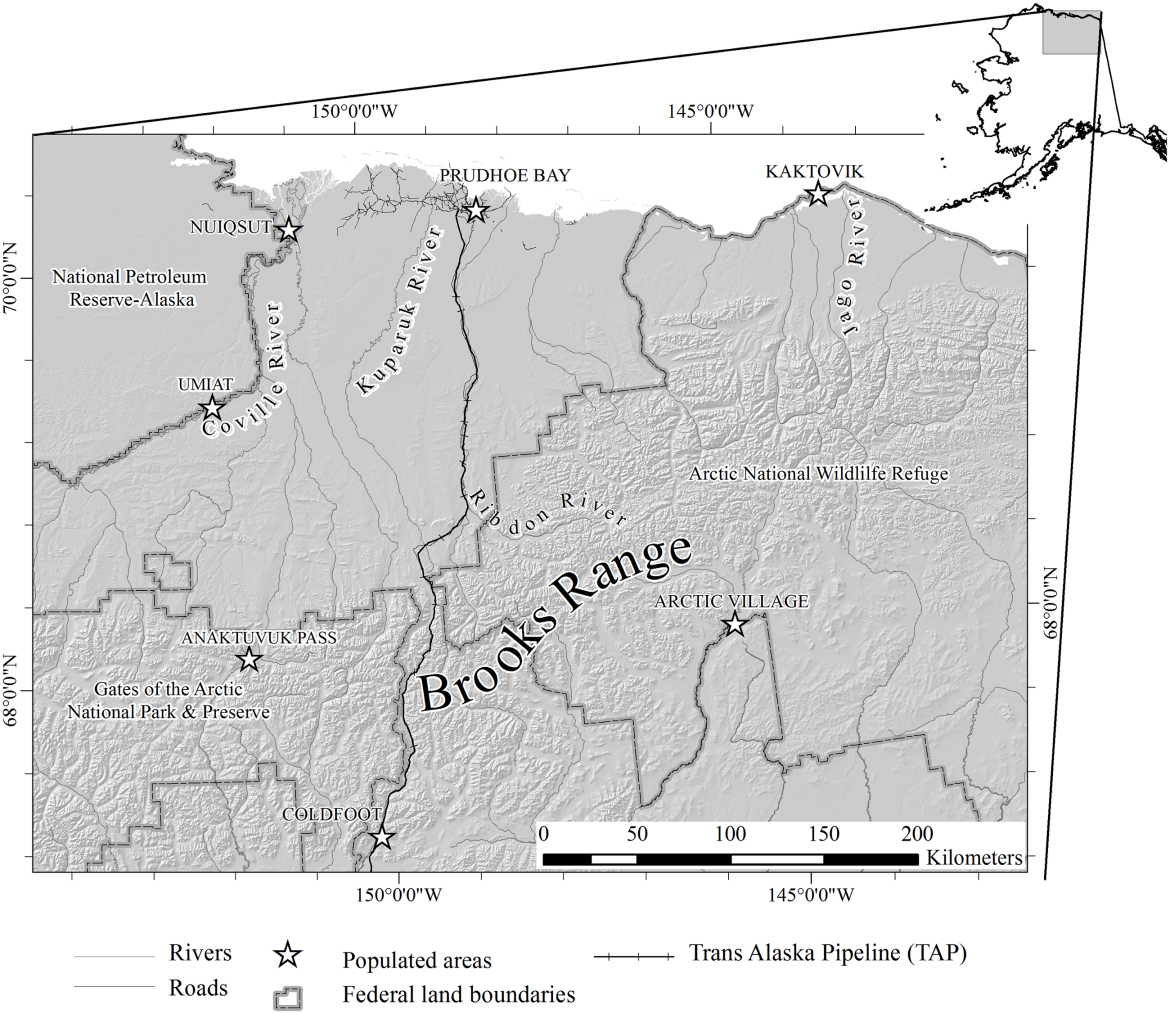
Study area in northern Alaska including the Dalton highway, the Trans-Alaska Pipeline (TAP), major land ownership divisions, and rivers mentioned in text.

The study area encompassed the annual range of the CAH, including wintering areas in the east-central Brooks Range and its northern foothills, migration routes, and calving and summer ranges on the coastal plain between the mountains and the Arctic coast (Fig 1). Climate varied greatly with latitude and elevation. Coastal areas were characterized by cool, moist summers and cold, dry winters, whereas inland areas were warmer during summer and similarly cold during the winter. Mean July temperatures ranged from 8 C along the coast to 13 C in the foothills and mountains, while mean February temperatures ranged from −29 C on the coast to −25 C in the southern Brooks Range. Mean monthly snow depths were greatest during February and March, ranging from 10 cm at Prudhoe Bay to 56 cm in the Brooks Range (Alaska Climate Research Center, http://climate.gi.alaska.edu/Climate/Location/Index.html). Snowmelt generally began during late April or early May and proceeded from south to north; most areas were snow free from early June through mid-September. The area includes lands administered by the State of Alaska, U.S. Bureau of Land Management, U.S. Fish and Wildlife Service–Arctic National Wildlife Refuge, and U.S. National Park Service–Gates of the Arctic National Park and Preserve.

The area was approximately bisected by the TAP corridor, which is oriented north–south and includes the Dalton Highway (Fig 1). The area west of the TAP corridor included the Prudhoe Bay industrial area, situated along the Arctic coast and extending westward to the Colville River delta. The area east of the corridor was largely undeveloped, except for relatively small industrial sites at Badami Point and Point Thomson, situated on the coast approximately 50 and 80 km east of Prudhoe Bay, respectively; a pipeline followed the coastline between Badami Point and Prudhoe Bay. The remainder of the area was remote and subject to little human activity except for dispersed recreation (hiking, river floating, and hunting).

Calving areas and much of the herd’s summer range were on the coastal plain, which was characterized by low relief and elevations ranging from sea level to 100 m. The coastal plain extended 35–95 km inland and included many shallow lakes and drained lake basins, ice-wedge polygons, scattered pingos, and river terraces. Vegetation communities were dominated by wet and moist graminoid tundra, with scattered patches of dwarf and taller shrubs, including willow (*Salix* spp.) and birch (*Betula nana* and *B*. *glandulosa*; [38, 51, 52]). Caribou sometimes used areas in the northern foothills of the Brooks Range during summer. These included rolling hills with elevations of 300–600 m, with vegetation consisting of graminoid meadows, dwarf shrub, and alpine tundra communities. Migration routes and wintering areas included rugged terrain, high peaks, and river valleys of the Brooks Range. Elevations ranged from 300–2,800 m. Land cover consisted of alpine tundra, rocky slopes, and permanent ice and snow fields at higher elevations, with graminoid meadows, shrub communities, and limited areas of spruce (*Picea mariana* and *P*. *glauca*) forest at lower elevations and along streams. Other ungulates and predators of the area included moose, muskoxen (*Ovibos moschatus*), grizzly bears (*Ursus arctos*), wolves (*Canis lupus*), wolverines (*Gulo gulo*), and golden eagles (*Aquila chrysaetos*).

### Caribou capture and monitoring

During March each year, 2003–2006, we captured adult female caribou using a hand-held net-gun fired from a low-flying helicopter (model R-44, Robinson Helicopter Co., Torrance, California). Procedures for handling live animals conformed to guidelines of the American Society of Mammalogists [53] and were approved by the Alaska Department of Fish and Game Animal Care and Use Committee. Captures occurred on State lands and permission for employees to capture animals on state lands is granted under the authority of Alaska Administrative Code 5AAC 92.033. Twenty-eight of these caribou had been radio-collared during previous years and were known to be ≥3 years old. We also captured 29 caribou of unknown age; in these cases we selected mature animals (based on body size, antler development, and tooth wear). Although most of these were likely ≥3 years old, it is possible that some 2-year-old caribou were included. Captured caribou were blindfolded and restrained by hobbles, examined briefly for injuries, and fitted with radio-collars. Handling time was limited to <10 min to minimize stress to the animal. We equipped caribou with radio-collars containing satellite-linked GPS receivers (model TGW3680, Telonics Inc., Mesa, Arizona) programmed to determine an animal’s position at intervals of 47 h during winter (Nov–Apr) and 5 h during summer (May–October). Location data were stored on-board the collars and relayed by satellite uplink using the Argos system (CLS America, Inc., Lanham, Maryland) once per week during winter and daily during summer. Collars contained a release mechanism programmed to detach the collars near the projected end of life of the batteries (2.5 years). However, we recaptured most caribou and replaced their radio-collars before the programmed release dates.

We downloaded data from recovered collars and used Tracking Analyst^®^ extension for ArcGIS software (version 9.3, ESRI, Redlands California) to plot locations of each individual. We screened the data and eliminated locations that were >100 km from the remaining locations. Using the tracking extension, we then examined the presumed path of the animal and identified unlikely movements as indicated by abrupt deviations from the general direction of movement, with an immediate return (three successive locations suggesting a back and forth movement with a speed >1.5 km/h; [54]. This procedure eliminated most cases where a single location was >500 m from the path indicated by a sequence of locations. We considered small spatial location errors to be acceptable for modeling large scale seasonal ranges and migration routes. We did not explicitly test accuracy of the GPS positions recorded by the collars. However, we visited 16 sites where collared caribou died during the study. We determined the locations of these sites with a hand-held GPS (Model 295, 495, or 60CSx, Garmin, Inc., Olathe, Kansas) and compared these with locations recorded by the GPS collars. Locations of all 16 caribou deaths as determined by handheld GPS units were ≤100 m from the locations recorded by the collars.

We created all maps in ArcGIS. All geospatial data used for the base maps was in the public domain and was obtained from the USGS national map (viewer.nationalmap.gov) or the Alaska State Geo-spatial Data Clearinghouse (www.asgdc.state.ak.us).

### Seasonal classifications

**Fig 2:**
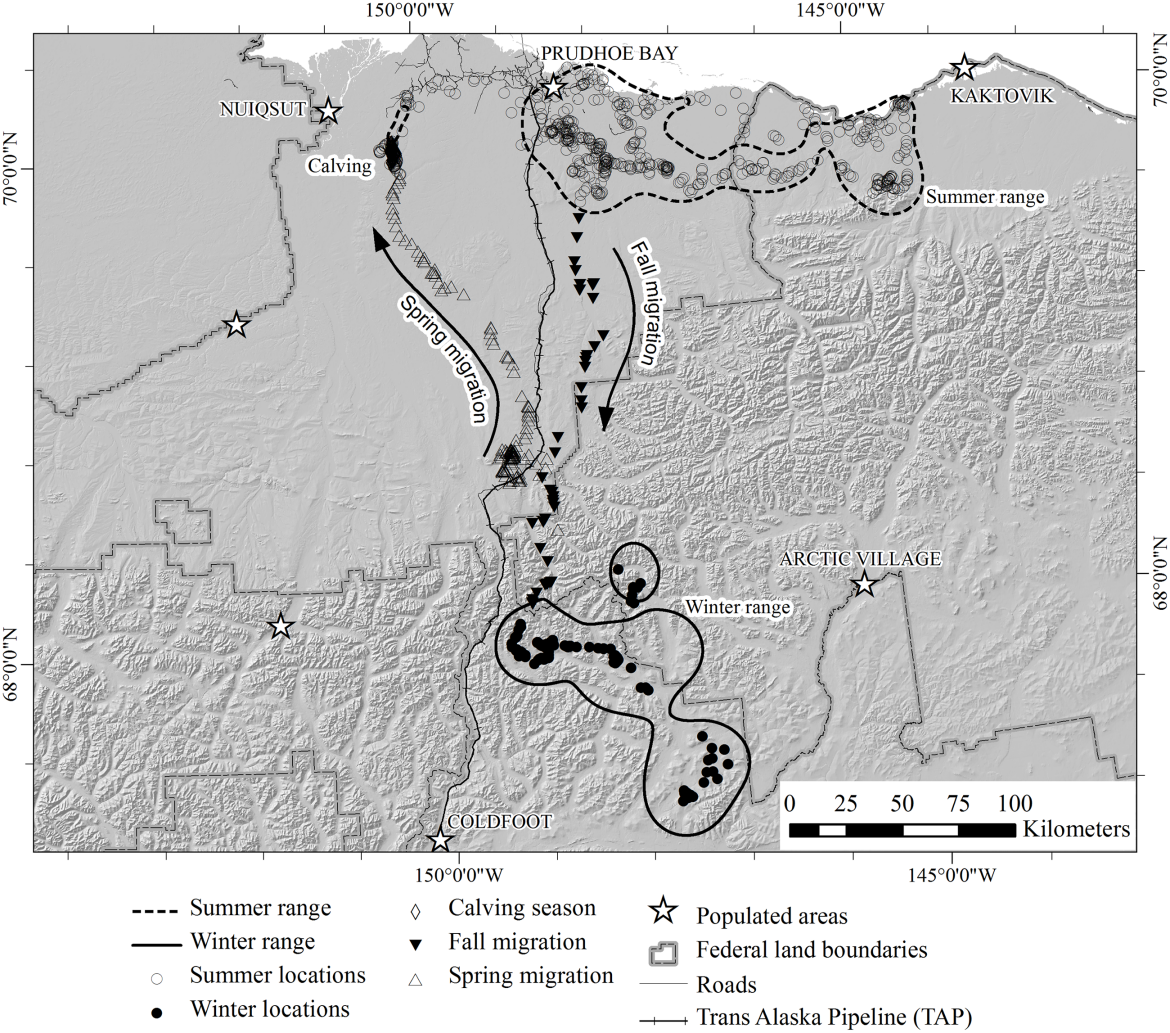
Example of seasonal movements of one caribou in northern Alaska during 2004. The caribou moved from the wintering area, followed the spring migration route to the calving area and summer range and then followed the fall migration route back to its winter range.

We categorized location data for each individual caribou into 5 periods (winter, spring migration, calving, summer, and fall migration). We did not model calving areas because these have been described elsewhere [28, 37, 38], but we considered calving as a separate season because movements of parturient caribou at this time were much different than during other seasons. Because of the variability in caribou movement patterns, we were unable to find a quantitative approach that would reliably distinguish migration periods from seasonal ranges (also see [55]). Instead, we classified location data into the aforementioned categories based on visual interpretation of the movement trajectories for each individual. We based these classifications on major differences in caribou movement patterns (primarily speed and consistency of direction) that were evident among seasons. As an initial step, we noted that previous studies indicated that caribou in northeastern Alaska usually began spring migration in March, April, or May [41, 43, 44, 46], calving occurred during the first 2 weeks of June [56–58]), and fall migration occurred during September–November [41, 43, 47]. We refined these periods for each individual based on an interpretation of their movement patterns by sequentially plotting their locations. We classified migration as a series of movements that were directed (i.e., following a consistent direction without significant backtracking) and oriented towards the general areas used during either summer or winter (i.e., heading north or northwestward during spring; or south or southeastward during fall; Fig 2 and S1 Video). Summer ranges were large, and caribou sometimes began fall migration by crossing a large portion of the summer range. Because our objective was to identify geographic areas used for migration routes as distinct from seasonal ranges, we limited overlap between summer ranges and fall migration routes by modeling the fall migration route for each individual beginning with the first location that was outside of the 80% isopleth of the summer range (see below). Spring migration began more abruptly, with little overlap of the winter range, so we were able to distinguish this behavior based solely on direction and speed of movement. For both spring and fall, we identified the end of migration as a change in behavior to much shorter movements with no obvious directional trend.

We defined winter as the period between the end of fall migration and the beginning of spring migration, and summer extended from the end of the calving period to the start of fall migration. We determined the calving season by observing collared caribou daily (weather permitting) during the first 2 weeks of June and determining when calves first were observed accompanying the collared animals [37]. Caribou that produced calves confined their movements to areas <1.5 km^2^ for periods of 10–14 days following the calf’s birth (this study, unpublished data). For most caribou, summer began when caribou moved beyond the small area used during calving. However, we observed 8 cases of collared caribou that did not produce calves or exhibit restricted movements during the calving period. For these animals, we modeled summer ranges beginning with the mean date of the end of calving by caribou that produced calves.

### Movement metrics

We estimated metrics to describe timing, and speed of migration. We calculated duration of migration as the number of days from beginning to end of an individual migration path and average velocity for vectors defined by each pair of consecutive locations, and then calculated the means of these over each individual migration path. Because the movement metrics were derived from consecutive points obtained at different intervals (due to failed fix attempts and different seasonal fix schedules), we corrected for the difference between the rates of acquisition. To do this, we converted all observed movement distances into what would be expected if locations had been taken at 5-h intervals by adding a correction factor (CF), so that:

$$Dist_{5\text{h}}\;=\;DistT\;+\;CF,$$

where *Dist*_*5h*_ was the corrected distance for a 5-h interval and *Dist*_*T*_ was the observed distance between two locations separated by a time interval of *T*. We determined the correction factor by comparing distances travelled along original tracks in which locations were obtained at 5-h intervals to distances measured between the endpoints of the same track after intervening locations had been removed. We removed locations to create intervals of 10–50 h between locations, increasing by 5-h increments. Based on all of our location data, the correction factors for spring and fall movements were:

$$CF_{\text{Spring}}\;=\;Exp\left[-0.94\;+\;2.36\;\times \;T\right],\;$$

and

$$CF_{\text{Fall}}\;=\;Exp\left[-1.10\;+\;2.42\;\times \;T\right].$$

By using this correction factor, we avoided subsampling data thus losing the resolution from the spring and summer when acquisition interval was more frequent than fall.

### Analysis and modeling

We used the Home Range Tools (HRT; [59]) extension for ArcGIS to model summer and winter ranges based on fixed-kernel density estimators, and used likelihood cross-validation (CVh) to choose the bandwidth [60]. Kernel home range models can be greatly affected by sample size and sampling frequency [61, 62]. To prevent under-smoothing of summer range models due to acquiring locations at high frequency (i.e., 1 every ~5 h) and to ensure comparable range estimates between seasons, we subset summer location data by systematically selecting locations that were separated by ≥47 h (i.e., we retained 1 of every 10 locations). Because sample sizes within a season for a given year were approximately equal among individuals, for each season and year, we pooled location data across individuals and defined population-level seasonal ranges as the isopleths encompassing 90% of the kernel-based utilization distributions (UD). For each year, we also determined the isopleth that encompassed 80% of the UD, which we used to delineate the beginning of the fall migration path.

We evaluated the extent of separation between seasonal ranges for each year by estimating the percent overlap between 90% UD ranges used during summer and the following winter. We examined fidelity of the herd to a particular seasonal range (i.e., summer or winter) in 2 ways. First, we estimated the percent overlap between ranges used in the same season during consecutive years. Second, we estimated the frequency of use (number of years) for areas within the composite seasonal ranges over the 4 years of the study. To do this, we used the union tool for ArcGIS to create 2 composite ranges, consisting of the 4 individual summer and winter ranges, respectively. Then, we calculated the percent of the total area of each composite that was used during each of the winter and summer seasons.

To delineate migration routes, we used the Brownian bridge movement model (BBMM; [63, 64]), which provides an estimate of the relative frequency-of-use (i.e., UD) of areas along the migration route for each individual. We modeled these routes using the BBMM package in R [65] using a grid-cell size of 500 m to balance biological significance, resolution of available geographic data, and to maintain reasonable processing time. Because this method models an animal’s movements along a sequence of locations, it is necessary to model each individual separately. Thus, similar to [64], we estimated the UD of the migration route for each caribou during each season of migration. We then modeled the population-level migration routes for spring and fall of each year as the mean of the individual UDs from that season [64].

Finally, we examined variation in migration routes used in different years by determining the amount of overlap among routes defined by the 95% isopleths of the population-level BBMMs. For each season, we overlaid the four annual routes and determined the number of years of use (potential range = 1–4 years) for all parts of the combined routes.

## Results

We collared a total of 57 female caribou and monitored them for periods of 3–52 months. Of these, 54 caribou provided sufficient data for modeling ≥ 1 seasonal range or migration path, with an overall fix success rate of 98% of 124,930 scheduled fixes. The lowest acquisition rate of any individual caribou was 76%; rates for all other caribou were >91%, suggesting that the data were not strongly biased by geographic features or other factors that might reduce fix acquisition rates.

We estimated summer ranges based on annual samples of 22–44 caribou (40–229 locations/caribou), and winter ranges based on annual samples of 18–44 caribou (48–298 locations/caribou). We estimated 106 spring and 88 fall migration paths involving 52 individual caribou. We observed 64 instances (25 and 39 for spring and fall, respectively; Table 1) where movements of a caribou during spring or fall did not fit our definition of migration; thus, we did not estimate migration paths for those caribou during those seasons. These caribou did not demonstrate a distinct migratory behavior pattern (i.e., they had overlapping summer and winter ranges and their movements between seasonal ranges were irregular and less directed).

**Table 1:** Numbers of migratory and non-migratory female caribou who met the definition of migration from the Central Arctic herd monitored in northern Alaska by season, 2003–2007.

	Fall					Spring				
	Migratory		Non-migratory			Migratory		Non-migratory		
Year	*n*	%	*n*	%	Total	*n*	%	*n*	%	Total
2003	18	78	5	22	23	19	83	4	17	23
2004	27	60	18	40	45	41	87	6	13	47
2005	23	77	7	23	30	23	70	10	30	33
2006	20	69	9	31	29	23	82	5	18	28
Total	88	69	39	31	127	106	81	25	19	131

**Fig 3:**
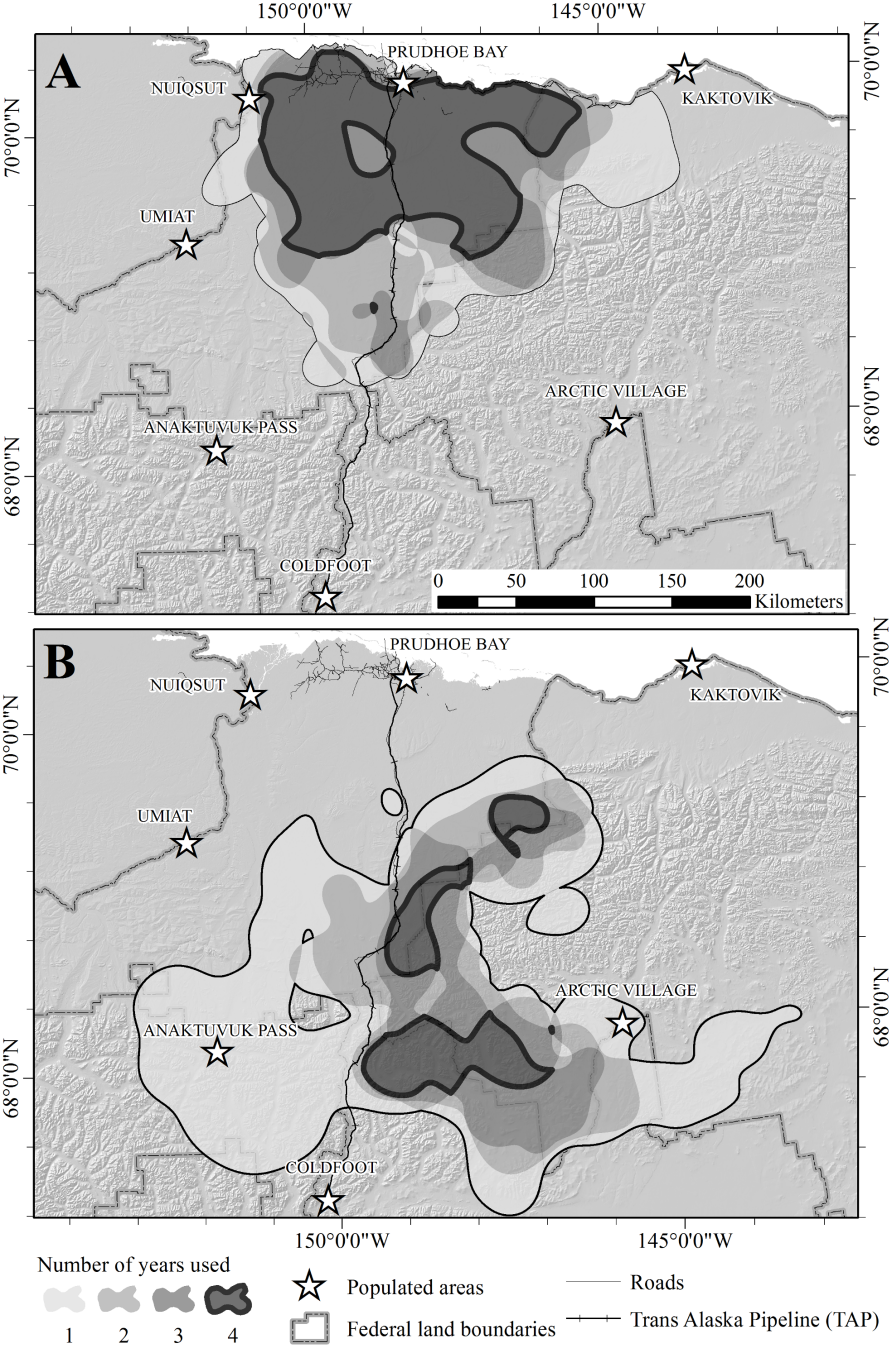
Years of use of seasonal ranges of the Central Arctic caribou herd in northern Alaska, 2003–2007. (A) summer ranges. (B) winter ranges. Ranges were modeled as the 90% isopleths of fixed-kernel utilization distributions for each season and year. Shading indicates the number of years each area was used (i.e., number of intersections of the 4 annual ranges modeled for each season).

**Fig 4:**
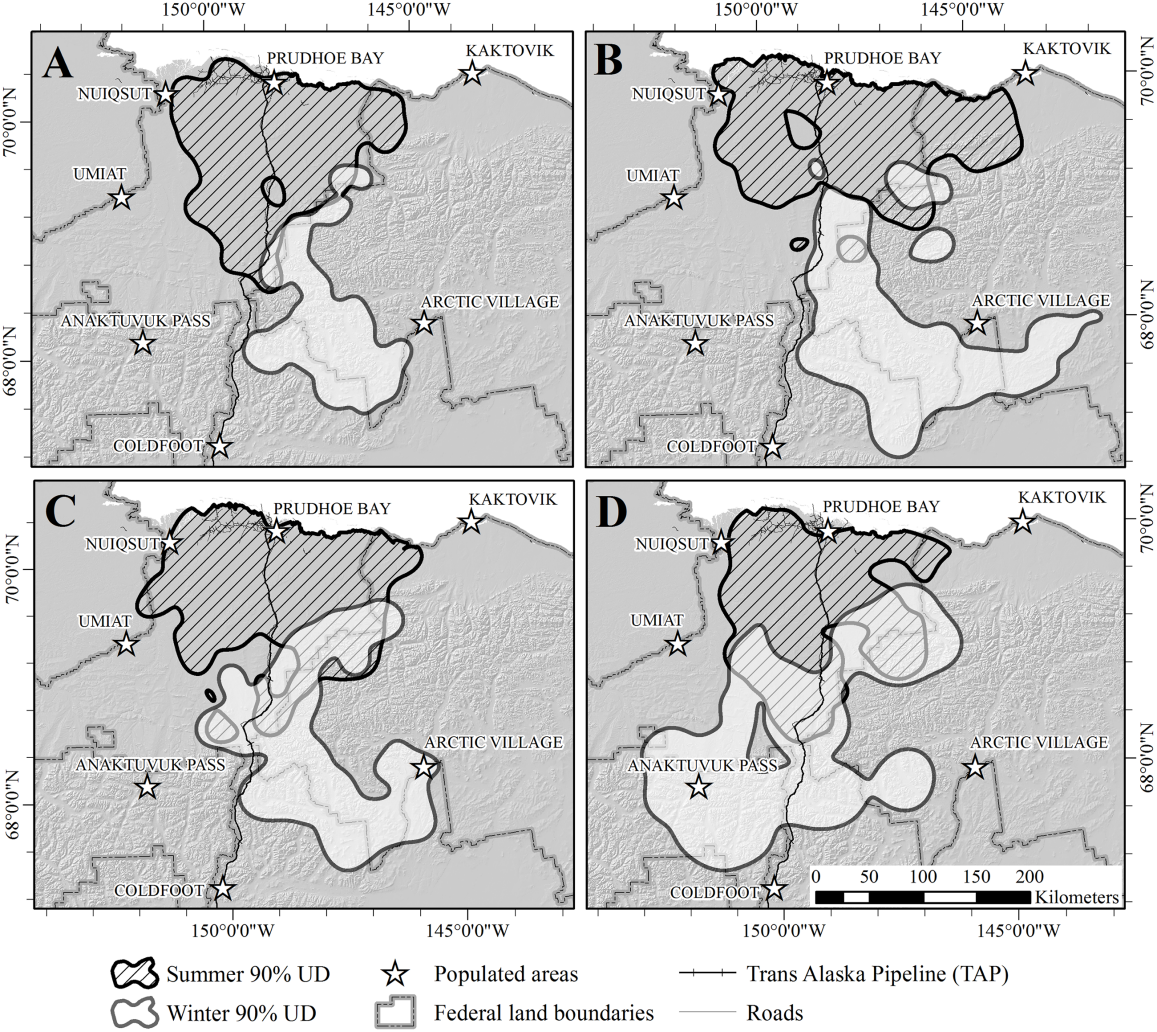
Annual estimates of seasonal ranges for the Central Arctic caribou herd in northern Alaska based on the 90% isopleths of fixed-kernel utilization distributions. (A) 2003–2004 (B) 2004–2005 (C) 2005–2006 (D) 2006–2007.

The combined area used during summer and winter for all years was 84,543 km^2^. The total area used for summer ranges encompassed 39,966 km^2^, whereas the total area used for winter ranges was 57,457 km^2^ (Fig 3). The area of overlap between combined summer and winter ranges was 14,781 km^2^ (17.5% of the total area used). Annual estimates of areas encompassed by the 90% UD for winter range (*X̅* = 26,585 km^2^; SE = 4,912 km^2^) were similar to estimates for summer range (*X̅* = 27,929 SE = 1,064 km^2^; *t*_*6*_ = 0.4493, *P* = 0.67). Within years, overlap between summer and winter ranges was 3.3, 3.8, 13.6, and 18.3% for 2003–2006, respectively (Fig 4). Within seasons, overlap between ranges used in consecutive years was less for winter (*X̅* = 42.8%, range = 31.3–50.9%) than for summer (*X̅* = 62.4%; range = 56.4–67.5%; *t*_*4*_ = 2.8994, *P* = 0.04). Only 10.7% of the total winter range was included in winter range models for every year, and 28.7% was used during ≥3 years, whereas 40.0% of the total summer range was included during every year and 60.9% was used during ≥3 years (Table 2; Fig 3).

**Table 2:** Percent of the total area of summer and winter ranges used during multiple years by caribou of the Central Arctic herd in northern Alaska, 2003–2006. Total area was the aggregate of 90% fixed kernel UDs estimated for all years (39,966 and 57,457 km^2^ for summer and winter range, respectively).

	Seasonal range	
Years used	Summer	Winter
1	21.0	54.3
2	17.8	16.9
3	20.9	18.1
4	40.0	10.7

**Fig 5:**
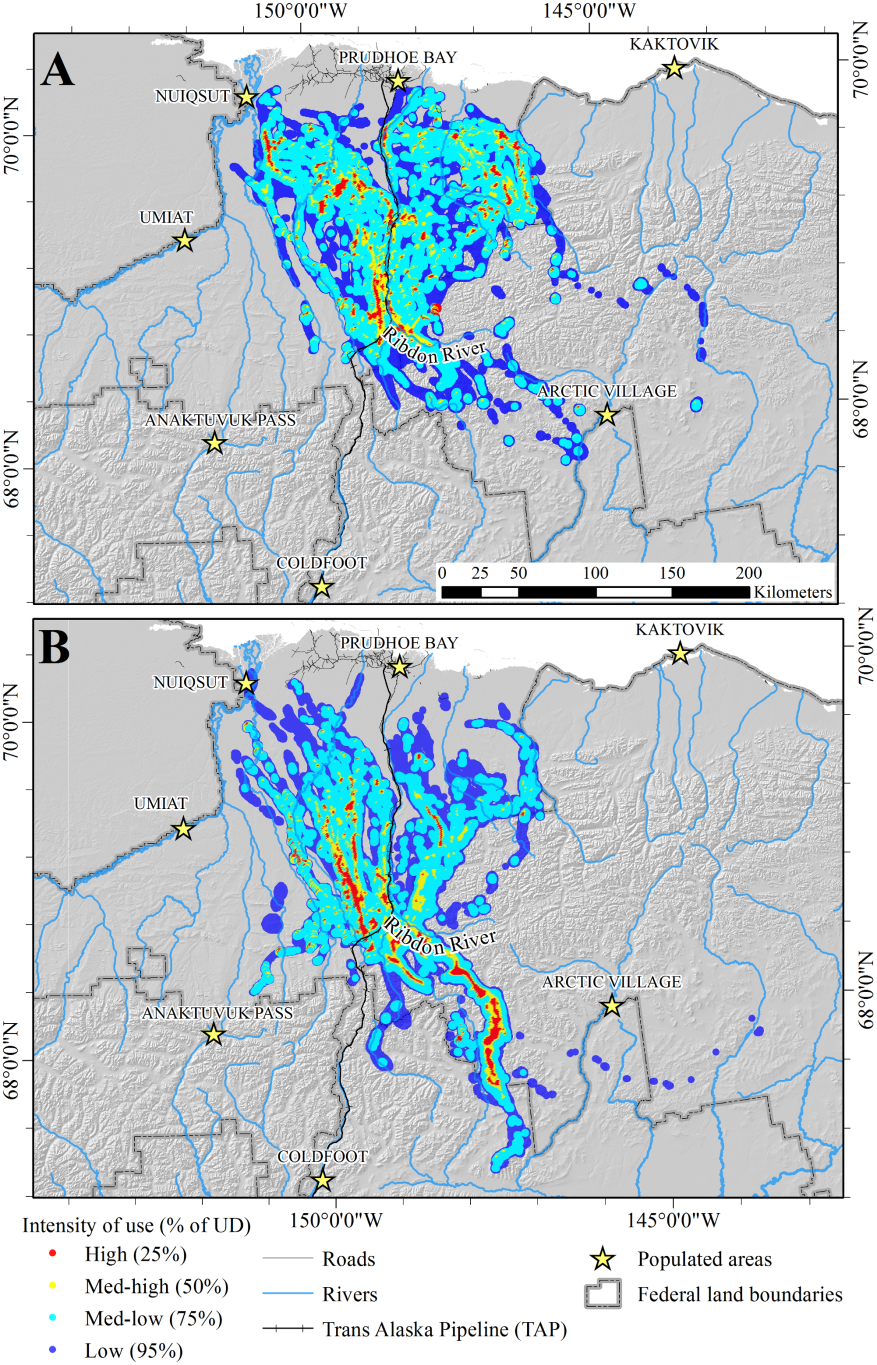
Relative use of migration routes by the Central Arctic caribou herd in northern Alaska, 2003–2007. (A) spring (B) fall. Use of 500-m grid cells was estimated as the mean of Brownian Bridge Movement Models for each year. Contours enclose portions of the UD with corresponding levels of use (i.e., 25% contour encloses 25% of the UD with the highest probability of use).

**Fig 6:**
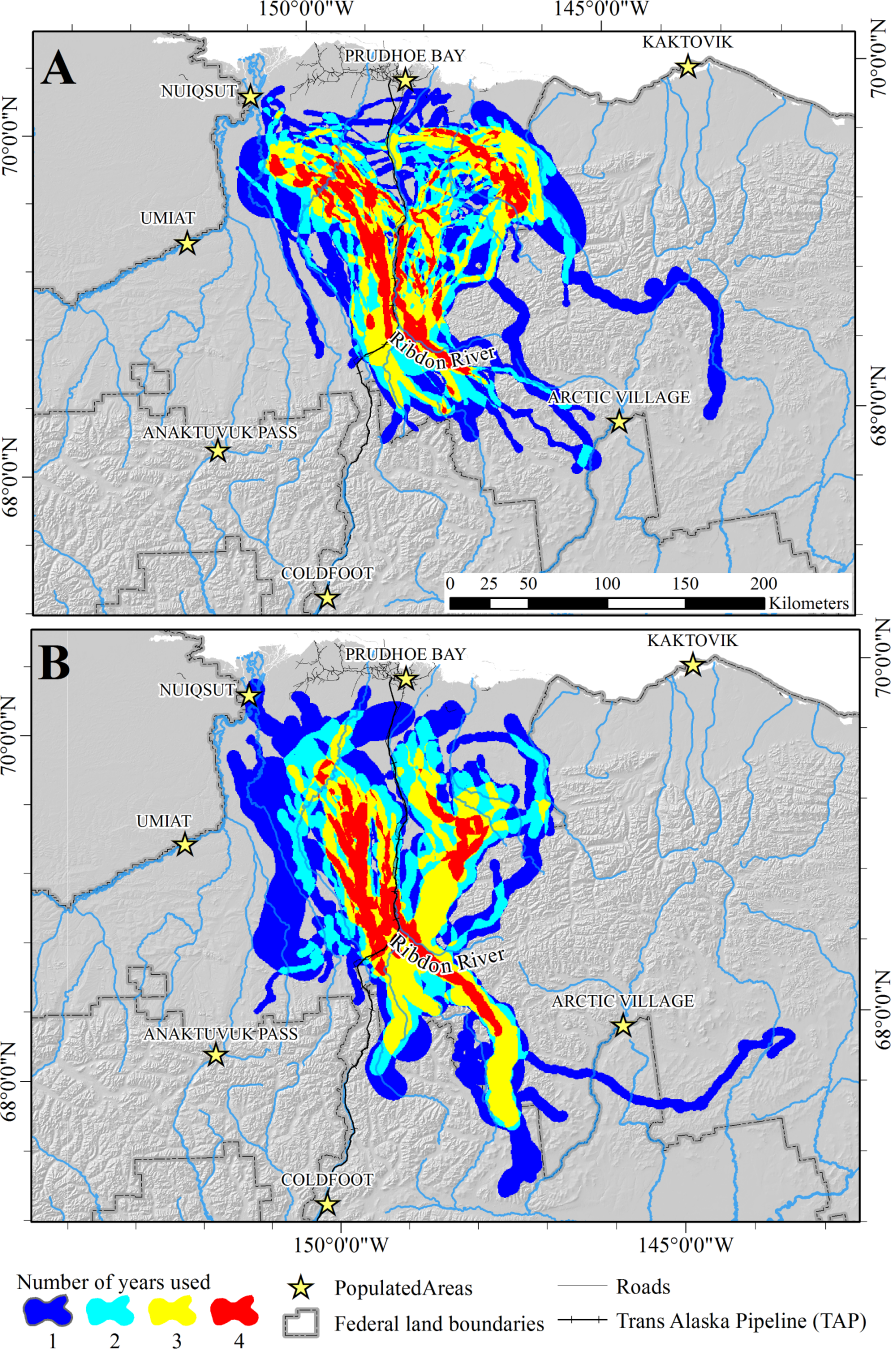
Number of years of use of seasonal migration routes by the Central Arctic caribou herd in northern Alaska, 2003–2007. (A) spring (B) fall. Each route was defined as the mean of the 95% isopleths of the Brownian Bridge Movement Models of all caribou for each season.

Caribou began spring migration near the beginning of May during all years (Table 3), and mean duration of migration was 14.72 days (Table 4). Fall migration was initiated in mid-September and mean duration was 12.54 days (Table 4). Migration routes estimated by averaging BBMMs of individual caribou overall years and by overlaying routes from different years produced similar results, and both methods indicated several areas of concentrated use (Figs 5 and 6).

**Table 3:** Mean annual start and end dates of seasons classified by caribou movement characteristics estimated for fall and spring migrations of the Central Arctic caribou herd in northern Alaska, 2003–2007. Summer extended from the end of calving to the beginning of fall migration; winter extended from the end of fall migration to the beginning of spring migration.

	Spring migration		Calving		Fall migration	
Year	Start	End	Start	End	Start	End
2003	11 May	26 May	2 Jun	13 Jun	11 Sep	20 Sep
2004	1 May	21 May	1 Jun	11 Jun	15 Sep	1 Oct
2005	29 Apr	17 May	1 Jun	13 Jun	30 Sep	14 Oct
2006	6 May	21 May	2 Jun	12 Jun	17 Oct	14 Nov
2007	5 May	4 Jun	4 Jun	9 Jun		

**Table 4:** Movement parameters, mean estimates, standard deviations (SD), and 95% confidence intervals (CI) and their mean annual start and end dates of seasons classified by caribou movement characteristics estimated for fall and spring migrations of the Central Arctic caribou herd in northern Alaska, 2003–2007. Sample sizes were the numbers of migration routes of individual caribou modeled during each season (88 and 106 for fall and spring, respectively). Summer extended from the end of calving to the beginning of fall migration; winter extended from the end of fall migration to the beginning of spring migration.

				95% CI	
Parameter	Season	Estimate	SD	Lower	Upper
Duration (days)	Fall	12.54	1.1	11.33	13.75
	Spring	14.72	1.1	13.52	15.92
Average velocity (m/5 h)	Fall	743.68	1.32	738.63	748.73
	Spring	496.05	1.29	491.1	500.99

## Discussion

Timing and distances moved by the CAH during spring migration were similar to those reported for the Porcupine herd [43, 44]. Our data also support the observations of Cameron and Whitten [47], who described the movements of the CAH during fall migration as more directed and rapid compared to spring migration. This difference might be partly due to differences in weather and environmental conditions, especially characteristics of the snowpack. During early spring, snow cover in northern Alaska is extensive and deep snow may restrict or slow caribou movements [19, 22]. During spring migration, caribou are in the poorest physical condition of the year and pregnant females face high energetic demands of gestation [66], which may further reduce their ability to travel rapidly. Conversely, fall migration typically occurs before deep snow has accumulated, and caribou movements are relatively unimpeded. Bergerud et al. [67] observed similar differences in movement rates between spring and fall migrations of caribou in eastern Canada, which they attributed to seasonal differences in food availability.

Most CAH caribou were migratory, and we found relatively little overlap between summer and winter ranges, especially considering that the 90% UD isopleths that we used to define ranges encompassed areas of relatively low use by caribou. Caribou whose movements did not fit our strict definition of migration also used different ranges between seasons, although these ranges often overlapped more than those of migratory caribou.

We observed considerable variably in range use despite only monitoring caribou for 4 years; we would likely have observed even greater variation had we considered a longer span of data. The greater variability in location of winter ranges vs. summer ranges suggests that caribou may have altered their use of winter range based on inter-annual differences in winter weather, snow cover, forage characteristics, or other factors. Historical data suggest that changes in winter distributions are typical of the CAH over long periods, and to some extent these changes parallel changes in herd size. Skoog [26] cited historical accounts from the late 19^th^ and early 20^th^ centuries describing periods of alternately high and low abundance of caribou in parts of the south-central Brooks Range currently used as winter range by the CAH. When the CAH was first identified during the late 1970s, herd size was estimated at 4,000–6,000 and winter range was thought to be primarily north of the Brooks Range [47]. By the early 1990s, the CAH had grown to approximately 20,000 caribou, and some of the herd had begun to winter in the southern Brooks Range [68]. During the 4 years of our study, herd size was estimated to be >32,000 caribou, of which 54–69% wintered south of the Brooks Range continental divide [37]. In 2008, the CAH was estimated at 66,772 caribou and an estimated 95% of the herd wintered in the southern Brooks Range [68]. Similar shifts in range use related to changes in caribou abundance have been reported elsewhere in Alaska [69] and Canada [67].

Long-term changes in climate are also likely to affect migratory patterns and create challenges to the management of migratory species [70, 71]. In the case of migratory caribou, the availability of highly-nutritious, new vegetation during spring coincides with the conclusion of spring migration, initiation of calving, and subsequent formation of large post-calving aggregations [72]. Thus, changes in temperature, precipitation, and environmental productivity that affect the emergence of new vegetation are likely to induce major range shifts during spring [4, 71, 73]. The variability we found in caribou winter range use suggests that caribou might change their use of winter range in response to changing climatic conditions. Such behavioral flexibility is likely to be a positive trait in the face of future energy development and potential climate- driven changes in caribou habitat and resources. Therefore, management efforts intended to identify and protect important caribou habitats should be sufficiently flexible to accommodate such changes in behavior. Additional studies are needed to assess the causes and significance of inter-annual changes in caribou distribution and to determine how much area is needed to enable caribou to select seasonal ranges based on varying environmental conditions (weather, forage abundance and quality, etc.).

Caribou movements during migration are likely influenced by the search for optimal forage or for conditions that favor travel, and availability of these conditions varies with snow conditions [74]. Although empirical evidence indicating the demographic consequences of fragmenting migration routes or converting them to unusable habitat is limited [75], increased development along caribou migration routes could have negative energetic effects if migratory movements are impeded or deflected to less-optimal areas. Effects of disturbance may be most important for female caribou, because increased energetic costs of migration may cause females to arrive in the calving areas in poor condition reducing the likelihood of survival of both the female and her offspring [56, 76].

Use of the BBMM enabled us to delineate population-level migration routes of the CAH, which may facilitate prioritizing areas for future management [64]. Perhaps due to the spatial variation in winter ranges, CAH caribou used multiple migration routes, or a network of corridors, rather than a single migration route. Migration paths crossed the TAP corridor in multiple locations, suggesting that mitigation measures incorporated into pipeline construction were largely successful in reducing the impact of the pipeline on caribou migrations. However, not all areas were used equally; some route segments were used by larger proportions of the CAH than others during both seasons (Figs 5 and 6), and the migration paths showed areas of high concentration, or bottlenecks. Migration bottlenecks typically occur where topography, vegetation, development, or other landscape features restrict animal movements to narrow or limited regions [77]. In particular, the concentration of migratory paths that crossed the Dalton Highway and TAP between the Kuparuk and Ribdon Rivers should be considered an important area for caribou migration when effects of future development proposals are evaluated. However, we do not recommend focusing management efforts solely on areas of high use, because areas used less frequently during one period may have high value at another time due to changes in vegetation, climate conditions, or disturbance regimes. As with seasonal ranges, geographic and temporal variation in migration routes must be considered for effective management of migratory caribou herds.

Because of the dynamic nature of caribou movements, identifying important areas or habitats used for particular behaviors is difficult and may be influenced by decisions regarding how to define specific movement patterns. For example, we found that caribou movements during migration were too variable to enable us to use a single model for identifying migratory movements of all individuals (e.g., [78]). Instead, we utilized multiple sources of information, including characteristics of the caribou movements themselves, to assign movements to different seasons [79, 80]. Although defining migration based largely on movement patterns of the caribou being studied was useful for identifying geographic areas used for this activity, such subjective decisions might influence the results of studies that attempt to identify factors that influence migration behavior or compare movements among different populations. However, changes in patterns of space use by animals in response to external stimuli can reveal underlying habitat requirements, energetic constraints, or other ecological relationships. Thus, studies that assess variation in movement patterns, rather than assuming consistency in range use and movements, can provide valuable insights into a species’ habitat relationships and management requirements.

## Supporting Information

S1 Video Animation of seasonal movements of one caribou in northern Alaska during 2004. The caribou moved from the wintering area from 2003/2004, followed the spring migration route to the calving area and summer range and then followed the fall migration route back to its 2004/2005 winter range.
(ZIP)
